# Barcoding intracellular reverse transcription enables high-throughput phenotype-coupled T cell receptor analyses

**DOI:** 10.1016/j.crmeth.2023.100600

**Published:** 2023-09-29

**Authors:** Sahana Jayaraman, Janelle M. Montagne, Thomas R. Nirschl, Emily Marcisak, Jeanette Johnson, Amanda Huff, Meng-Hsuan Hsiao, Julie Nauroth, Thatcher Heumann, Jelani C. Zarif, Elizabeth M. Jaffee, Nilo Azad, Elana J. Fertig, Neeha Zaidi, H. Benjamin Larman

**Affiliations:** 1 Institute for Cell Engineering, Division of Immunology, Department of Pathology, Johns Hopkins School of Medicine, Baltimore, MD 21205, USA; 2 Department of Oncology, Sidney Kimmel Comprehensive Cancer Center, Johns Hopkins University School of Medicine, Baltimore, MD 21205, USA; 3 Convergence Institute, Johns Hopkins University School of Medicine, Baltimore, MD 21205, USA; 4 Bloomberg Kimmel Immunology Institute, Johns Hopkins University School of Medicine, Baltimore, MD 21205, USA; 5 Pathobiology Graduate Program, Department of Pathology, Johns Hopkins University School of Medicine, Baltimore, MD 21205, USA; 6 Bloomberg∼Kimmel Institute for Cancer Immunotherapy, Johns Hopkins Medicine Sidney Kimmel Comprehensive Cancer Center, Baltimore, MD 21205, USA; 7 Department of Genetic Medicine, Johns Hopkins University School of Medicine, Baltimore, MD, USA; 8 Department of Immunology, Johns Hopkins University School of Medicine, Baltimore, MD 21205, USA; 9 Division of Hematology Oncology, Vanderbilt-Ingram Comprehensive Cancer Center, Vanderbilt University Medical Center, Nashville, TN, USA; 10 Division of Quantitative Sciences, Department of Oncology, Sidney Kimmel Comprehensive Cancer Center, Johns Hopkins University School of Medicine, Baltimore, MD 21205, USA; 11 Department of Applied Mathematics and Statistics, Johns Hopkins University, Baltimore, MD 21218, USA; 12 Department of Biomedical Engineering, Johns Hopkins University School of Medicine, Baltimore, MD 21205, USA

**Keywords:** repertoire sequencing, antigen receptors, adaptive immunity, mutant KRAS

## Abstract

Assays linking cellular phenotypes with T cell or B cell antigen receptor sequences are crucial for characterizing adaptive immune responses. Existing methodologies are limited by low sample throughput and high cost. Here, we present INtraCEllular Reverse Transcription with Sorting and sequencing (INCERTS), an approach that combines molecular indexing of receptor repertoires within intact cells and fluorescence-activated cell sorting (FACS). We demonstrate that INCERTS enables efficient processing of millions of cells from pooled human peripheral blood mononuclear cell (PBMC) samples while retaining robust association between T cell receptor (TCR) sequences and cellular phenotypes. We used INCERTS to discover antigen-specific TCRs from patients with cancer immunized with a novel mutant KRAS peptide vaccine. After *ex vivo* stimulation, 28 uniquely barcoded samples were pooled prior to FACS into peptide-reactive and non-reactive CD4^+^ and CD8^+^ populations. Combining complementary patient-matched single-cell RNA sequencing (scRNA-seq) data enabled retrieval of full-length, paired TCR alpha and beta chain sequences for future validation of therapeutic utility.

## Highlights

• INCERTS uses intracellular barcoding of cDNA prior to pooled cell sorting
• Upstream sample pooling efficiently links immune repertoires with cell phenotypes
• INCERTS readily scales to millions of cells from a large number of samples

## Motivation

Existing methodologies that integrate T or B cell phenotypes with their antigen receptor sequences are limited by low sample throughput and high cost. Such approaches include single-cell RNA sequencing (scRNA-seq) and flow cytometric sorting of cells into subpopulations for bulk repertoire sequencing, both of which are difficult to scale across many samples and millions of cells. Therefore, we developed INtraCEllular Reverse Transcription with Sorting and sequencing (INCERTS). INCERTS successfully links cellular phenotype with antigen receptor sequence via barcoding during reverse transcription within intact cells. Subsequent cell sorting, sequencing, and demultiplexing enables high-throughput characterization of phenotype-coupled antigen receptor sequences across millions of cells.

## Graphical abstract

**Figure:**
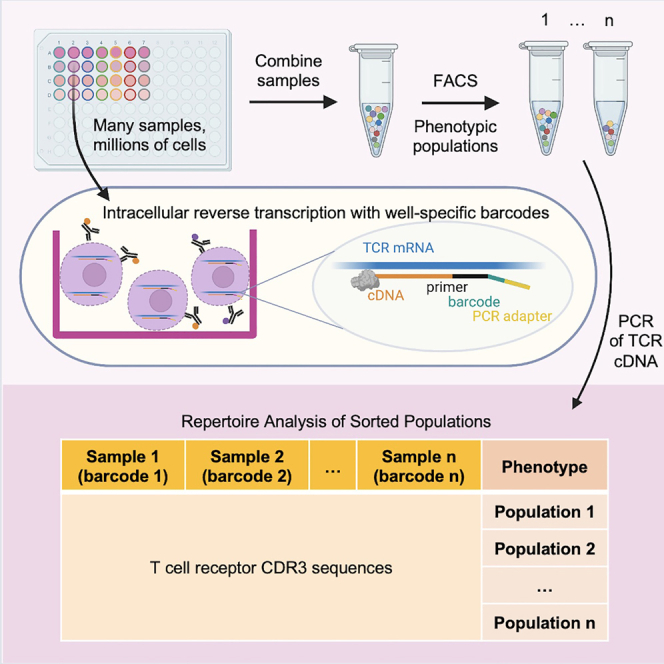

## Teaser

Jayaraman et al. develop INCERTS, a scalable and efficient method for linking cell phenotypes to immune receptor sequences. INCERTS uses intracellular cDNA barcoding, which enables pooling of samples prior to cell sorting, thereby linking cell phenotypes with immune receptor repertoire sequences.

## Introduction

T cells recognize their target antigens via diverse repertoires of T cell receptor (TCR) sequences. The characterization of adaptive immune responses thus frequently involves defining the TCR encoding sequences present in blood or tissue.^1^^,^^2^^,^^3^^,^^4^ The TCRβ chain confers much of the antigen specificity to a T cell, and its complementarity-determining region 3 (CDR3), which is the product of VDJ combinatorial and junctional diversity, is often used to uniquely define T cell clonotypes. Notably, integrating TCR sequencing with functional or phenotypic measurements, such as antigen stimulation and subsequent flow cytometric analysis, can reveal key features of adaptive immune responses.^5^^,^^6^^,^^7^

Current methods for linking TCRs to cellular phenotype can be grouped into two main categories: (1) single-cell RNA sequencing (scRNA-seq) and (2) flow cytometric sorting of cells into subpopulations for bulk repertoire sequencing. scRNA-seq enables simultaneous analysis of mRNA and protein expression within individual cells (i.e., via upstream sorting or multiomics analyses such as Cellular Indexing of Transcriptomes and Epitopes, or CITE-seq^8^) and can define the paired alpha and beta TCR sequences necessary for reconstruction of a functional receptor. However, these approaches are limited in throughput, for example ∼10^4^ cells per sample using currently available single-cell technologies. Furthermore, the cost to scale these experiments to millions of cells, which may be necessary for certain applications, including identification of rare antigen-specific clones, may be prohibitive. Alternatively, millions of cells can be analyzed at a relatively low per-cell cost using phenotypic cell sorting and subsequent analysis via bulk TCR sequencing. These approaches are therefore complementary, and their integration could provide a deeper sampling of the repertoire with broad cellular phenotyping and paired TCRα/β sequencing.

Despite having a relatively high cellular throughput versus scRNA-seq, fluorescence-activated cell sorting (FACS)-based separation of cellular subpopulations is somewhat limited in sample throughput. Further, separately sorted samples may suffer from imperfect comparability.^9^ For example, 30 samples sorted into four subpopulations each will result in a cumbersome 120 samples that require individual downstream processing prior to bulk sequencing. To overcome these limitations, we developed INtraCEllular Reverse Transcription with Sorting and sequencing (INCERTS) to increase the sample throughput and sampling depth of phenotype-coupled TCR repertoires. Upstream sample multiplexing is accomplished via intracellular reverse transcription (RT) of TCRβ mRNA molecules using DNA-barcoded primers. In this way, numerous barcoded samples can be pooled together upstream of sorting into phenotypic subpopulations for downstream repertoire sequencing. TCRβ sequences are mapped back to samples of origin via their sample-specific barcodes. In the previous example of 30 samples and four subpopulations of interest, the pooled barcoded samples can be sorted into just 4 subpopulations (rather than 120) for bulk repertoire sequencing. Thus, INCERTS greatly simplifies the workflow of otherwise overly complex experimental designs.

Here, we demonstrate the utility of INCERTS for identifying potentially rare cancer-specific TCRs using peripheral blood mononuclear cells (PBMCs) from a set of patients receiving a novel mutant KRAS peptide vaccine. We find 119 candidate mutant KRAS-reactive TCRs. Furthermore, we show how integration of INCERTS with complementary patient-matched scRNA-seq data can be used to recover full-length alpha and beta chain sequences for 65 TCRs (including five of 119 candidate KRAS-reactive TCRs). Notably, INCERTS can be extended beyond T cells and TCRs to facilitate experiments requiring the analysis of millions of cells from large numbers of samples.

## Results

**Figure 1:**
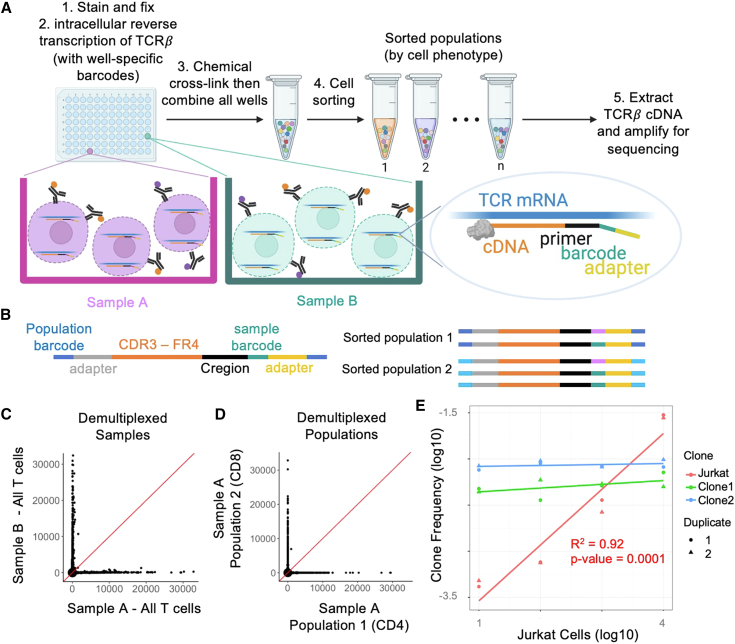
INCERTS methodology (A) Schematic of INCERTS protocol. Samples A and B are shown in two different wells, where they are stained with two different fluorescently conjugated antibodies. Each sample contains TCRβ cDNAs generated from RT primers with sample-specific barcodes. (B) Annotated example PCR amplicons from two different sorted populations. FR4, framework region 4. (C and D) Sample-demultiplexed INCERTS data from a sorted pool of two donors, A and B (C), and cell population-demultiplexed INCERTS data from a single donor (D). Each dot corresponds to a unique CDR3 amino acid sequence, and values correspond to total clonal sequencing read count. (E) Jurkat cells spiked in at different input amounts into a background of PBMCs, in duplicate. Each point corresponds to a clonal frequency; Jurkat frequencies are shown in red, and the top two most abundant PBMC clone frequencies are shown in blue and green. R^2^ and p value of linear regression of Jurkat cell clone frequencies versus spike-in inputs are in red.

Figure 1A illustrates a typical INCERTS workflow. First, samples are stained with fluorescently conjugated antibodies to label surface proteins of interest. Stained cells are then fixed and permeabilized with methanol, which largely preserves the integrity of mRNA.^10^ Each sample is then combined with a TCRβ RT primer containing a unique sample-specifying barcode. After RT, cells are cross-linked with dithiobis(succinimidyl propionate) (DSP) to improve intracellular retention of cDNA during sorting. Samples are then mixed and sorted into populations of interest based on their labeled surface markers. TCRβ cDNA is next extracted from each sorted population and amplified with Framework Region 3 AmplifiKation sequencing (FR3AK-seq)^11^ primers and subpopulation-specific barcodes. In this way, TCRβ sequences in the final sequencing library carry both sample-specific barcodes and cell subpopulation-specific barcodes (Figure 1B). Barcode demultiplexing then maps each TCR sequence to a unique cell population and the sample from which it originated (Figures 1C and 1D). In a test of barcode fidelity, INCERTS was performed on T cells from two different donors, which were mixed and sorted into CD4^+^ and CD8^+^ T cell subpopulations. We observed negligible barcode exchange between samples or cell subpopulations (Figures 1C and 1D). These data suggest that INCERTS can be used to analyze hundreds of samples and millions of cells in a single flow sort with negligible barcode misassignment. To assess assay sensitivity and linearity, we spiked increasing numbers of clonal Jurkat T cells into a background of one million PBMCs (Figure 1E). Jurkat clonal frequency was linearly proportional to cellular input (linear regression R^2^ = 0.92 and p value = 0.0001). At the lowest level of ten spiked-in Jurkat cells, the Jurkat CDR3 sequence was detectable above background. The top two most frequent PBMC T cell clonal frequencies were constant across the Jurkat titration.

There is rapidly growing interest in the discovery of typically rare tumor-specific TCRs for therapeutic applications, and INCERTS is designed to screen the millions of cells from multiple samples required for this type of analysis. Therefore, we assessed the utility of INCERTS for identifying tumor-reactive TCRs directly from patients receiving a novel mutant KRAS peptide vaccine. Mutated KRAS makes up about 85% of all RAS mutations and has been previously associated with clonal expansion of anti-tumor T cells in human patients. RAS mutations are found in approximately 30% of all cancers and in over 90% of pancreatic cancers.^12^^,^^13^^,^^14^^,^^15^^,^^16^^,^^17^ Activating KRAS mutations are highly conserved and tend to occur at restricted amino acid positions. TCRs that recognize dominant KRAS mutants, whether to endogenous protein or peptide vaccine,^18^^,^^19^^,^^20^ are thus potential candidates for cellular therapies.^17^^,^^18^^,^^19^^,^^20^^,^^21^^,^^22^^,^^23^^,^^24^^,^^25^^,^^26^^,^^27^ We therefore used INCERTS to identify KRAS-reactive TCRs from individuals with pancreatic or colorectal cancer who were immunized with a mutant KRAS peptide vaccine (ClinicalTrials.gov: NCT04117087).

**Figure 2:**
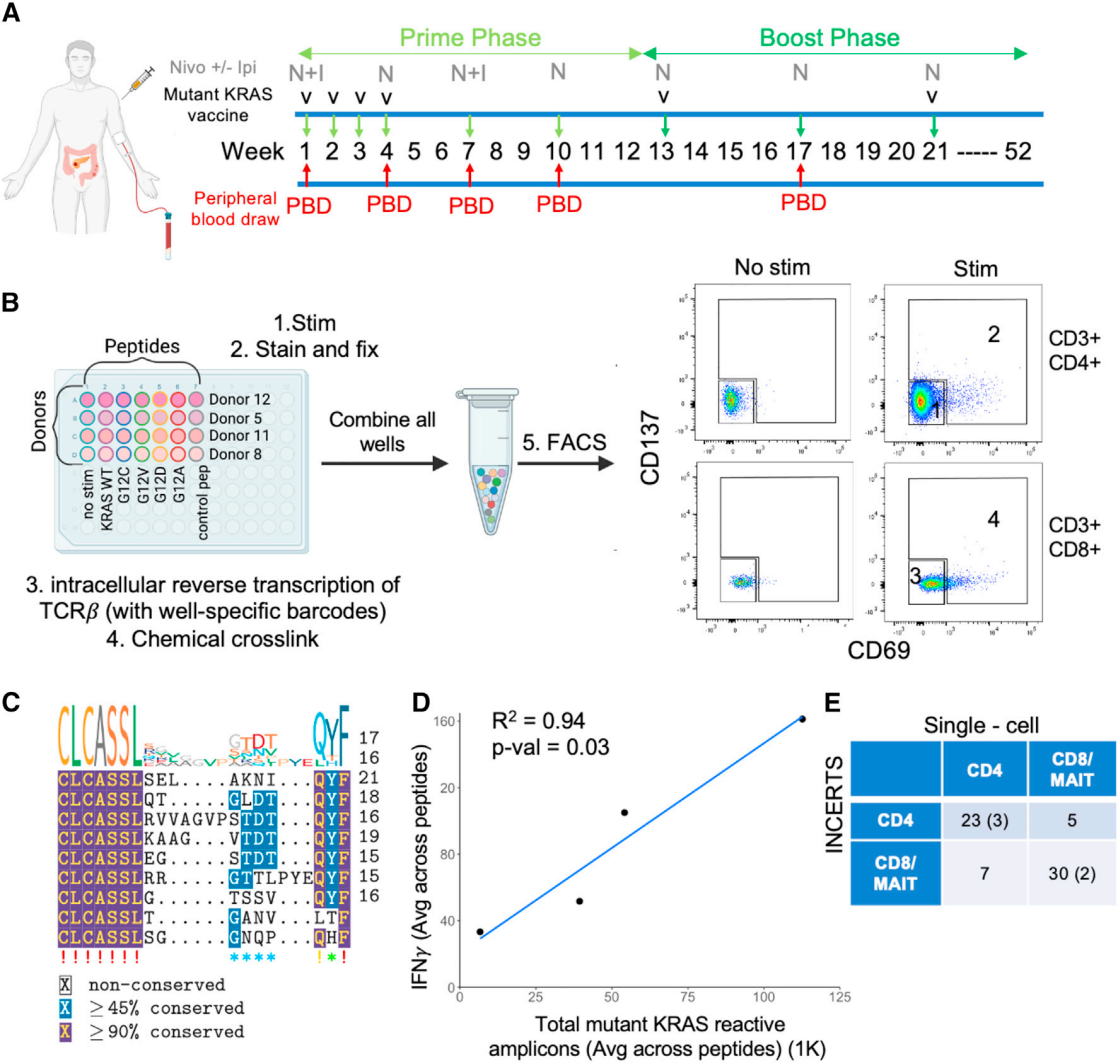
INCERTS identifies mutant KRAS-reactive TCRs from vaccine trial patients (A) Timeline of vaccine, immunotherapy, and peripheral blood draws is shown for patients enrolled in the mutant KRAS peptide vaccine trial.^28^ Nivo, nivolumab; Ipi, ipilimumab. (B) PBMCs from four different donors were each split into six different peptide stimulation conditions: wild-type KRAS peptide, four mutant KRAS vaccine peptides (G12C, G12V, G12D, and G12A), a control EBV peptide, and a no-peptide mock stimulation condition. All 28 stimulated cultures underwent the INCERTS protocol, were combined into a single pool, and then were sorted into four cell subpopulations. CD3^+^CD4^+^ and CD3^+^CD8^+^ population gating for CD69 and CD137 activation markers is shown. (C) Consensus motif and sequence alignment of mutant KRAS-reactive CDR3 sequences. See also Table S2. (D) Average number of total productive KRAS-reactive sequences identified via INCERTS (x axis) is plotted against the average frequency of IFNγ-secreting cells detected via ELISPOT (y axis) with R^2^ and p value of linear regression displayed. (E) Contingency table showing correspondence between INCERTS-assigned cell subpopulation versus scRNA-seq-assigned cell subpopulation for CDR3s detected in donor 12 by both methods. Peptide-reactive sequences are in parentheses. See also Tables S1, S3, and S4.

The mutant KRAS peptide vaccine comprises a mixture of six 21-amino-acid-long synthetic peptides encompassing common KRAS hotspot mutations in pancreatic and colorectal cancer—five alterations at the G12 position and one alteration at the G13 position. The dosing regimen includes both prime and boost phases, with concomitant anti-PD-1 or anti-CTLA4 immune checkpoint inhibitor therapy (Figure 2A). Peripheral blood draws were performed at the indicated time points. Samples from time points at which T cells demonstrated peptide reactivity by interferon γ (IFNγ) ELISPOT were prioritized for INCERTS analysis. PBMCs from each of the four donors (two with pancreatic cancer and two with colorectal cancer) were split into six separate peptide stimulation cultures each: four with individual mutant KRAS peptides (four of six mutant KRAS peptides were used due to limited sample availability, of which three corresponded to the most reactive peptides by ELISPOT [G12C, G12V, G12A] and one corresponded to the most reported TCR target [G12D]), one with a wild-type (WT) KRAS peptide, one with an Epstein-Barr virus (EBV) peptide, and one negative control culture without any peptide stimulation (Figure 2B). After 72 h, the 28 cultures were stained for CD3, CD4, and CD8 as well as for the T cell activation markers CD69 and CD137. Cells then underwent methanol fixation, reverse transcription, and DSP cross-linking as outlined in Figure 1A. The 28 samples were then mixed for a single FACS separation into four cell subpopulations: CD4^+^ or CD8^+^ T cells that were either activated (peptide reactive) or not activated based on CD69 and/or CD137 positivity (Figure 2B). cDNA was subsequently extracted from these four phenotypic populations, PCR amplified using FR3AK-seq primers and phenotypic barcodes,^11^ and then sequenced on a single Illumina flow cell.

Overall, INCERTS identified 186 productive, peptide-reactive TCRβ clones from the 28 stimulation conditions. 119 (64%) were mutant KRAS peptide reactive (Table S1), 24 (13%) were WT KRAS peptide reactive, and 43 (23%) were EBV reactive. Of these 186 reactive TCR sequences, 114 were from 8,206 activated CD4^+^ T cells and 72 of were from 7,170 activated CD8^+^ T cells (Table S4), in line with the ability of synthetic long peptides to induce both robust CD4^+^ and CD8^+^ T cell responses.^29^^,^^30^^,^^31^^,^^32^ From among the 349,713 not activated CD4^+^ T cells and the 73,269 not activated CD8^+^ T cells, 2,242 and 866 unique, productive TCRβ sequences, respectively, were also identified (Table S4).

Fifteen TCRβ clones were reactive to at least two peptides (Table S1)—ten from CD4^+^ sorted T cell populations and five from CD8^+^ sorted T cell populations. Five of these fifteen TCRβ clones were reactive against multiple mutant KRAS peptides. These five multireactive sequences (four from CD4^+^ cells and one from CD8^+^ cells) may have greater therapeutic potential. Although CD8^+^ T cell-derived TCRs are most commonly nominated as therapeutic candidates, CD4^+^ T cell-derived TCRs are increasingly proposed as viable therapeutic candidates.^33^^,^^34^^,^^35^ In comparison, four of the fifteen cross-reactive TCRβ clones appeared to be reactive to both WT and mutant KRAS peptides, thus limiting their therapeutic potential.

Analysis of mutant KRAS peptide-reactive TCRβs using the GLIPH2 software^36^ identified 21 convergent clusters. The most statistically significant cluster featured a conserved motif with N-terminal CLCASSL and C-terminal QYF (Figure 2C). CASSL with QYF is also found in published KRAS-reactive TCRβs (Table S3).^18^^,^^21^^,^^25^^,^^37^ Interestingly, this motif was detected in sequences from both CD4^+^ and CD8^+^ T cell populations from two different donors. Although CD4^+^ and CD8^+^ TCRβs are typically non-overlapping due to their distinct restrictions, studies have reported sequence similarities in CD4^+^ and CD8^+^ TCRβs in autoimmune,^38^ viral,^39^ and cancer^40^ patient populations. Notably, one sequence in this cluster is reactive to the control EBV peptide, and another sequence in this cluster is reactive to the WT KRAS peptide. Furthermore, all clusters containing TCRβ clones reactive to multiple mutant KRAS peptides also contained an EBV or a WT KRAS-reactive TCRβ clone, suggesting that these TCRs may be promiscuous in antigen specificity (Table S2). Because PBMC time points were selected for INCERTS analysis based on T cell reactivity to mutant KRAS by IFNγ ELISPOT, we next examined ELISPOT concordance with the INCERTS findings. We found that donors with more mutant KRAS-peptide-reactive T cells by ELISPOT also had more mutant KRAS-reactive TCRs detected via INCERTS (Figure 2D) (linear regression R^2^ = 0.94, p value = 0.03).

PBMCs from donor 12 were also analyzed using combined scRNA-seq and TCR-seq (10× Genomics). We therefore queried this complementary dataset to find TCRβ CDR3 sequences that matched INCERTS-identified sequences. Altogether, we found 65 matching TCRβ CDR3 nucleotide sequences. Of these, 56 (86.2%) had a TCRα chain that was sufficiently defined for functional reconstruction of the TCR. Notably, all 65 (100%) J gene sequences matched between the two datasets, as expected based on the location of the RT and FR3AK-seq primers used in INCERTS analysis. Furthermore, 53 of the 65 overlapping TCRβs (81.5%) were identically annotated as CD4^+^ or CD8^+^ in both datasets (Figure 2E). Five of the 65 overlapping TCRβs were identified by INCERTS as potentially mutant KRAS reactive (Table S3). Of these five sequences, one TCR with TRBV28 and TRBJ2-7 shares the same V/J pairing with a previously published mutant KRAS-reactive TCR.^37^ Another TCR with TRAV4 and TRAJ15 overlaps with V and J genes reported for four published mutant KRAS-reactive sequences (Table S3).^21^ Since the scRNA-seq data were generated from whole PBMCs, we expected that enrichment for T cells upstream of scRNA-seq would increase the number of overlapping TCRβs available for comparison. Indeed, these findings underscore the complementary nature of INCERTS and scRNA-seq, as the former enables identification of many candidate mutant KRAS-reactive TCRs, while the latter provides detailed phenotypic information and paired alpha/beta TCR sequences. Overall, these findings demonstrate the accuracy and utility of INCERTS to identify potentially neoantigen-specific TCRs. When integrated with patient-matched scRNA-seq data, these TCRs may be fully reconstructed for functional validation.

## Discussion

Fewer than 50 KRAS-specific TCRs have been reported in the literature to date.^18^^,^^21^^,^^25^^,^^37^^,^^41^^,^^42^ However, using INCERTS, 119 candidate mutant KRAS-reactive TCRβs, none of which overlap with known TCRs, were identified in a single experiment. Of these, 25 were reactive to G12C, 26 to G12V, 47 to G12D, and 21 to G12A. Notably, TCRβs specific for G12A are not yet reported in the literature, while G12V- and G12D-specific TCRβs are the most common. Additionally, only 1 of the established mutant KRAS-reactive TCR sequences was obtained from a patient with pancreatic cancer, while we report 38 candidates from two patients with pancreatic cancer. We further report 81 candidate KRAS-reactive TCRβs from two patients with colorectal cancer. Additionally, we identified a shared motif (beta chain) and shared V/J usage (alpha and beta chains) between our candidate mutant KRAS-reactive TCRs and published mutant KRAS-reactive TCRs.

Other methods have been developed to define antigen-specific TCRs linked to cellular phenotypes. Traditionally, antigen-specific populations have been identified by activation marker-positive populations, multimer positivity, or carboxyfluorescein succinimidyl ester (CFSE) dilution followed by bulk RNA-seq of individual populations.^43^ One such approach, MIRA,^44^ enables identification of many antigen-TCR pairs. However, each stimulation condition requires sorting into multiple populations, limiting the method’s scalability. An alternative approach, MANAFEST,^45^ relies on expansion of antigen-specific T cells that are quantified by bulk sequencing. A notable limitation of this approach is the inability to link TCRs with cellular phenotype. INCERTS’s intracellular barcoding strategy can be incorporated into the experimental setup of assays such as MIRA or MANAFEST, increasing the number of antigen stimulations and samples that can be processed simultaneously and efficiently. More recent methods for identifying TCR antigen specificity utilize scRNA-seq, enabling simultaneous detection of phenotype and paired alpha-beta TCRs. For example, SELECT-seq^46^ is a method that sorts single T cells after antigen stimulation. TCRs are sequenced to identify expanded clones, which are then analyzed individually via scRNA-seq. Although this method reduces the cost of sequencing by limiting transcriptomic analysis to clones of interest, the total number of T cells examined is limited to hundreds to low thousands. Another recently developed technology, TetTCR-seq HD,^47^ sorts on tetramer-positive populations prior to scRNA-seq. While tetramer-positive populations reveal antigen-specific TCRs, this approach relies on predefined epitopes. Furthermore, the use of tetramers requires a high-affinity TCR interaction, which may miss important lower-affinity TCRs.^48^ Other methods successfully linking protein-level phenotypic and transcriptomic information from immune cells, including CITE-seq,^8^ INs-seq,^49^ and CLInt-seq,^50^ have also been reported. Each of these use scRNA-seq for readout, making them expensive and constrained in the number of antigen-reactive clones assessed per experiment.

Overall, INCERTS enables sample mixing for efficient cell sorting and phenotype-embedded repertoire sequencing. In this proof-of-concept study, we used INCERTS to discover over 100 candidate mutant KRAS-reactive TCR sequences from a set of patients receiving a mutant KRAS peptide vaccine. Quick, inexpensive INCERTS-based quantification of specific TCRs or motif-containing TCRs across numerous patient samples and/or over multiple time points may also be used to track specific immune responses. Additionally, INCERTS can be applied for comparison of repertoires from stimulated and sorted populations to repertoires from millions of unstimulated and unsorted cells for direct quantification of clonal enrichment. In future studies, researchers may readily adapt the method to any markers of interest (e.g., to memory populations, exhausted populations, etc.). INCERTS could also be applied to B cells to identify B cell receptor (BCR) sequences in populations of interest (e.g., defined by phenotypic markers or even antigen-bound BCRs). Importantly, INCERTS may be extended beyond immune cells and their antigen-specific receptors to identify differences in cell populations with and without specific sequence variants or transcriptional patterns. INCERTS is thus a simple approach to link proteomic and transcriptomic information from hundreds of samples comprising millions of cells in a single integrated workflow.

### Limitations of the study

Some limitations in our experimental setup likely contributed to lower detection sensitivity for reactive TCRs. One PBMC sample per donor was split into multiple wells with one million cells each. Given that antigen-specific T cells post-vaccine can occur as infrequently as 1 in 100,000,^51^ relevant but rare clones may not be detected when stimulating small cultures that do not deeply sample the population. Furthermore, CD69^+^ cells were the largest fraction of our peptide-activated T cell population. Although CD69 is considered an early activation marker, its expression at later time points varies by assay. At around 72 h, it is possible that some T cells stimulated within the first 24 h of plating lost CD69 expression and thus were not sorted with the activated population. A further limitation of INCERTS is that it requires methanol fixation, which alters the availability of some protein epitopes and thus requires staining with antibodies before fixation. It therefore also requires the use of methanol-resistant fluorophores. However, if isolating only one T cell population, such as CD8^+^ T cells, INCERTS should also be compatible with magnetic sorting. In the current study, the cellular phenotypes used for sorting were limited to cell surface markers. While not yet tested, we expect that the protocol will also be compatible with staining of some intracellular proteins.

Our specific proof-of-concept experimental application resulted in repertoire analysis of activated CD4^+^ and CD8^+^ populations consisting of ∼15,000 cells total. While this cell number falls within a range that can be processed via scRNA-seq, INCERTS can be easily scaled to analyze many more samples (e.g., 100s) and many more cells (e.g., millions). Crucially, these large-scale analyses can be performed efficiently and at low cost using INCERTS. Furthermore, INCERTS data lack transcriptome information or paired immune receptor chain sequences. However, we demonstrate that INCERTS-identified TCRβ CDR3 sequences can be queried in patient-matched scRNA-seq data to recover full-length paired TCRs, highlighting the complementary nature of these technologies.

## STAR★Methods

### Key resources table

**Table:** 

REAGENT or RESOURCE	SOURCE	IDENTIFIER
**Antibodies**		

BV605 mouse anti-human CD4 (clone RPA-T4)	BD	Cat. No. 562658; RRID: AB_2744420
Alexa Flour 700 mouse anti-human CD8 (clone RPA-T8)	BD	Cat. No. 561453; RRID: AB_10643765
Alexa Flour 647 mouse anti-human CD3 (clone UCHT1)	BD	Cat. No. 557706; RRID: AB_396815
BV786 mouse anti-human CD69 (clone FN50)	BD	Cat. No. 563834; RRID: AB_2738441
BV421 mouse anti-human CD137 (clone 4B4-1)	BD	Cat. No. 564091; RRID: AB_2722503

**Biological samples**		

PBMCs from patients receiving mutant KRAS peptide vaccine	Johns Hopkins NCT04117087 clinical trial sample bank	N/A

**Chemicals, peptides, and recombinant proteins**		

RPMI media	ThermoFisher	Cat. No. 11875093
2-Mercaptoethanol	ThermoFisher	Cat. No. 21985023
GlutaMAX	ThermoFisher	Cat. No. 35050061
Antibiotic Antimycotic solution	MilliporeSigma	Cat. No. A5955
sterile-filtered, heat inactivated human AB serum	MilliporeSigma	Cat. No. H3667
IL2 cytokine	PEPROTECH	Cat. No., 200-02
1X PBS -Ca-Mg	Corning	Cat. No. 21-040-CV
Mutant KRAS 21-mer synthetic long peptides with the following mutations: G12C, G12V, G12D, G12A	This paper	N/A
Zombie green live/dead stain	BioLegend	Cat. No. 423111
Brilliant Stain Buffer	BD	Cat. No. 563794
Dithiobis (succinimidyl propionate) (DSP)	ThermoFisher	Cat. No. 22585
DL-Dithiothreitol (DTT)	Sigma-Aldrich	Cat. No. D9163
1% low melting point agarose	Invitrogen	Cat. No. 16520
CEF peptides used in ELISpot assay	CTL	Cat. No. PA-CEF-002
Fetal bovine serum	Hyclone	Cat. No. SH30071.03

**Critical commercial assays**		

SuperScript IV First-Strand Synthesis System kit for reverse transcription	ThermoFisher	Cat. No. 18091050
*Quick*-DNA/RNA Microprep Plus kit for cDNA extraction	Zymo	Cat. No. D7005
KAPA2G Fast Multiplex Kit for PCR	Roche	Cat. No. 07961430001
Herculase Fusion DNA Polymerase kit for PCR	Agilent	Cat. No. 600677
Monarch Gel Extraction kit	NEB	Cat. No. T1020S
ELISpot assay	Mabtech	3420-4HST
Chromium™ Single Cell 5′ Library & Gel Bead Kit v2	10x Genomics	Cat. No. PN-1000263
TCR Amplification kit	10x Genomics	Cat. No. PN-1000252

**Deposited Data**		
scRNA-seq with matched TCR-seq	This paper	dcGaP No. phs003425.v1.p1

**Oligonucleotides**		

1MM FR3AK-Seq multiplex PCR primer set for TCRβ chain	Montagne et al.^11^	N/A

**Software and algorithms**		

cutAdapt	Martin et al.^52^	https://cutadapt.readthedocs.io/en/stable/installation.html
MiXCR (version 3.0.13)	Bolotin et al.^53^	https://mixcr.com/mixcr/getting-started/installation/
GLIPH2	Huang et al.^36^	http://50.255.35.37:8080/
CellRanger (version 5.0.1)	N/A	https://support.10xgenomics.com/single-cell-gene-expression/software/pipelines/latest/installation
Scanpy (version 1.8.1)	Wolf et al.^54^	https://scanpy.readthedocs.io/en/stable/installation.html
Scrublet	Wolock et al.^55^	https://github.com/swolock/scrublet
Besca	Mädler et al.^56^	https://github.com/bedapub/besca
Azimuth	Hao et al.^57^	https://azimuth.hubmapconsortium.org/
Celltypist	Domínguez Conde et al.^58^	https://www.celltypist.org/
Scirpy (version 0.9.1)	Sturm et al.^59^	https://github.com/scverse/scirpy

**Other**		

96 well V-bottom plate	Corning	Cat. No. 3894
Protector RNase Inhibitor	Roche, Millipore Sigma	Cat. No. 333540200
sparQ PureMag beads	Quantabio	Cat. No. 95196-005
human T activator CD3/CD28 Dynabeads	ThermoFisher	Cat. No. 11131D

### Resource availability

#### Lead contact

Further information and requests related to protocol details, resources and reagents should be directed to the lead contact, H. Benjamin Larman (hlarman1@jhmi.edu).

#### Materials availability

This study did not generate new unique reagents.

### Experimental models and study participant details

#### Human participants

PBMCs from patients who received a novel mutant KRAS peptide vaccine are part of the on-going clinical trial NCT04117087: https://www.clinicaltrials.gov/study/NCT04117087. Johns Hopkins Medicine institutional permission and oversight has been granted via IRB00210915.

**Table:** 

	Study Population (n = 4)
Sex	
Male	3 (75%)
Female	1 (25%)
Age (in years)	
Mean	54.25 ± 10.2
Median	8
Range	50.5
Race	
Hispanic or Latino	0 (0%)
Other	1 (25%)
White	3 (75%)

Because this study only used four different donors, no conclusion can be made about the influence of sex and/or gender on these results.

### Method details

#### Peptide stimulation of PBMCs

PBMCs were rapidly thawed at 37°C and added dropwise to a 15 mL conical tube containing 1 mL of 37°C RPMI (ThermoFisher, Cat. No. 11875093). 6 mL of 37°C RPMI was then added slowly to wash the cells, followed by centrifugation at 1200 rpm for 10 min. The supernatant was discarded, and cells were resuspended in 5 mL 37°C complete media (RPMI + 1X BME (Thermofisher, Cat. No. 21985023) + 1X GlutaMAX (Thermofisher, Cat. No. 35050061) + 1X antibiotic antimycotic solution (MilliporeSigma, Cat. No. A5955) + sterile-filtered, heat inactivated human AB serum at 10% v/v (MilliporeSigma, Cat. No. H3667)) and centrifuged at 1200 rpm for 10 min to wash, repeated for a total of two washes. The supernatant was discarded, and cells were resuspended in 10 mL complete media (1x10^6^ cells/mL) and transferred to a 10 cm dish to rest for at least 3 h at 37°C and 5% CO_2_.

After resting, 200 μL PBMCs were plated at 5x10^6^ cells/mL per well, with 100IU IL2/mL (PEPROTECH, Cat. No., 200-02) and 2 μg peptide/mL in a 96 well V-bottom plate (Corning, Cat. No. 3894). Mutant KRAS 21-mer synthetic long peptides with the following mutations were used for stimulations: G12C, G12V, G12D, G12A. Positive control EBV peptide and KRAS WT peptide were used at the same concentration. 1 μL DMSO/mL (matching the volume of peptide added) was used for the unstimulated (no peptide) condition. Cells were incubated at 37°C and 5% CO_2_ for ∼72 h.

#### Cell staining

200 μL of Jurkat cells (to be used as a spike-in post cell-sorting) were added to 1 well of the V-bottom plate (Corning, Cat. No. 3984) at 5x10^6^ cells/mL to be stained along with stimulated PBMC samples. The V-bottom plate containing all samples was then centrifuged at 400xg for 10 min to pellet cells, and the supernatant was discarded by gently flicking the plate. To wash, cells were resuspended with 200 μL of 1X PBS -Ca-Mg (Corning, Cat. No. 21-040-CV) plus 0.05U/μL of Protector RNase Inhibitor (Roche, Millipore Sigma, Cat. No. 333540200) (PBS-RI) and centrifuged at 400xg. This was was repeated for a total of two washes. Cells were then resuspended in 1:1000 Zombie green live/dead stain (BioLegend, Cat. No. 423111) in 50 μL PBS-RI for 20 min at room temperature in the dark. Cells were then washed with 150 μL PBS-RI and centrifuged at 400xg for 10 min. The supernatant was discarded, and cells were resuspended in 55 μL Brilliant Stain Buffer (BD, Cat. No. 563794) with 0.2U/μL of Protector RNase Inhibitor, for 15 min at room temperature in the dark with the following antibody dilutions: 1:100 CD4 RPA-T4 clone in BV605 (BD, Cat. No. 562658), 1:250 CD8 RPA-T8 clone in AF700 (BD Cat. No. 561453), 1:50 CD3 clone UCHT1 in AF647 (BD, Cat. No. 557706), 1:500 CD69 clone FN50 in BV786 (BD, Cat. No. 563834), and 1:100 CD137 clone 4B4-1 in BV650 (BD, Cat. No. 564091). Cells were washed with 150 μL PBS-RI and centrifuged at 400xg for 7 min. Three additional washes were performed with 200 μL PBS-RI and centrifugation at 400xg for 7 min. Cell pellets were resuspended in 35 μL of PBS-RI.

#### Cell fixation with methanol

160 μL of 100% ice-cold molecular biology grade methanol was added dropwise to each well (final 80% v/v methanol). Cells were then mixed by gentle pipetting up and down after all methanol addition. The V-bottom plate was placed at 4°C for 15 min in the dark. 3 μL of 5% Triton X-100 in nuclease-free water was added to each well to augment cell pelleting, and the plate was centrifuged at 400xg for 10 min at 4°C. Cells were subsequently washed two more times with 200 μL of 0.001% Triton X-100 in PBS-RI and 400xg centrifugations for 7 min at 4°C. Stained and fixed cells were then resuspended in 10 μL of PBS-RI.

#### INCERTS reverse transcription (RT)

Each well of methanol-fixed cells was pre-annealed with RT primer for 1 h at room temperature, in the dark as follows: 10 μL of cells in PBS-RI with 2 μL of 2 μM well-specific barcode primer, 8 μL of 5X buffer from SuperScript IV First-Strand Synthesis System kit (ThermoFisher, Cat. No. 18091050), 5 μL of 1:10 diluted Protector RNase Inhibitor and 15 μL of nuclease free water. After pre-annealing, 150 μL of 0.001% Triton X-100 in PBS-RI was added to cells. The plate was centrifuged at 400xg for 7 min 4°C. Supernatant was removed and cells were washed once more with 200 μL of 0.001% Triton X-100 in PBS-RI followed by centrifugation at 400xg for 7 min at 4°C.

Cell pellets were resuspended with SSIV reverse transcription master mix containing 4 μL 5X RT buffer, 1 μL 10 mM dNTP mix, 1 μL 0.1M DTT, 1 μL RNase inhibitor, 1 μL SSIV reverse transcriptase, 0.13 μL Protector RNase inhibitor, and 10.9 μL nuclease free water. Samples were transferred to 200 μL thin-walled PCR tubes and incubated at 50°C for 10 min for reverse transcription. After RT, cells were transferred back to the V-bottom plate. 175 μL of 0.001% Triton X-100 in PBS-RI was added to cells and they were resuspended by gentle pipetting. Cells were centrifuged at 400xg for 7 min at room temperature. Supernatant was removed and the plate was washed once more in 200 μL 0.001% Triton X-100 in PBS-RI and centrifuged at 400xg for 7 min at room temperature.

#### DSP cross-linking

Cell pellets were resuspended in 200 μL of 0.25 mg/mL Dithiobis (succinimidyl propionate) (DSP) (ThermoFisher, Cat. No. 22585) in 1X PBS with 0.2 U/μL of Protector RNase Inhibitor for 30 min at room temperature in the dark. 5 μL of 1 M Tris (pH 7.5) was added to each well to quench reactions at a final concentration of 20 mM Tris for 10 min. The plate was then centrifuged at 400xg for 7 min at room temperature and supernatant was discarded. Cells were washed twice with 200 μL of 0.001% Triton X-100 in PBS-RI and centrifuged for 10 min, discarding supernatant each time. The Jurkat cells were resuspended in 200 μL of PBS-RI, transferred to a 1.5 mL DNA LoBind tube, and stored at 4°C until after FACS of PBMCs. Remaining cell pellets were resuspended in 50 μL of PBS-RI each. All cells were then transferred into a single tube for FACS analysis.

#### Cell-sorting and cDNA extraction

Sorting was performed on a FACSAria Fusion (4-way sort) into activated and not activated populations for both CD4 (CD3^+^CD4^+^) and CD8 (CD3^+^CD8^+^) cells. The activated population was defined as CD69^+^CD137^+/−^ and CD69^+/−^CD137^+^. The not activated population was CD69^−^CD137^−^. A negative control (no peptide) sample was used to determine gating. Samples were sorted into PBS-RI in 1.5 mL DNA LoBind tubes. After the sort, the volumes of inactivated populations were brought up to 500 μL by adding the necessary volume of PBS-RI, and the volumes of activated populations were brought up to 200 μL. 10000 Jurkat cells stored after DSP cross-linking were added to each tube as a monoclonal spike-in. All tubes were centrifuged at 400xg for 10 min at room temperature. Supernatant was removed from each tube such that 100 μL was remaining, to preserve the cell pellet. All samples were incubated in 50 mM of DL-Dithiothreitol (DTT) (Sigma-Aldrich, Cat. No. D9163) (5.25 μL of 1M DTT to 100 μL of sample) for 10 min at room temperature to reverse the DSP crosslinks. 100 μL of DNA/RNA Lysis Buffer was added to each tube containing 100 μL of sample (1:1 ratio of lysis buffer to sample) and purification proceeded as per *Quick*-DNA/RNA Microprep Plus kit instructions (Zymo, Cat. No. D7005). Samples were eluted in 22 μL of nuclease free water and stored at −20°C until PCR amplification.

#### Amplification and sequencing

8 μL of cDNA from each sorted population was amplified via 20 cycles of PCR using the KAPA2G Fast Multiplex Kit (Roche, Cat. No. 07961430001) and the 1MM FR3AK-Seq multiplex primer set,^11^ with each forward primer at 0.00835 μM and the reverse primer at 0.125 μM. PCR conditions were as follows: 1) 95°C for 3 min, 2) 95°C for 15 s, 47°C for 30 s, and 72°C for 30 s for 20 cycles, 3) 72°C for 1 min. A second PCR was performed to add cell subpopulation-barcoded forward and reverse primers using 2 μL of PCR1 product and primers at 0.25 μM each with the Herculase Fusion DNA Polymerase kit (Agilent, Cat. No. 600677). PCR conditions were as follows: 1) 95°C for 2 min, 2) 95°C for 20 s, 58°C for 20 s, and 72°C for 30 s for 20 cycles, 3) 72°C for 3 min. Barcoded samples were then mixed together and an equal volume of sparQ PureMag beads (Quantabio, Cat. No. 95196-005) were added. Bead clean-up proceeded as per manufacturer’s instructions. Briefly, samples and beads were mixed together until homogeneous, incubated at room temperature for 5 min then placed on a magnet. Supernatant was removed and samples were washed twice with 80% ethanol (30 s each time) while still on the magnet. Beads were then dried and eluted in nuclease free water. The pooled sample was assessed on a 1% low melting point agarose (Invitrogen, Cat. No. 16520) gel in 1X TAE buffer and then gel extracted per Monarch Gel Extraction kit protocol (NEB, Cat. No. T1020S). Sequencing was performed on an Illumina NextSeq with 150bp single-end reads.

#### Jurkat spike-in experiment

INCERTS was performed as described above, with the following modifications. CD14^−^ PBMCs were stimulated for 2.5 days with human T activator CD3/CD28 Dynabeads (ThermoFisher, Cat. No. 11131D). 1x10^6^ Dynabead stimulated PBMCs were aliquoted per well, into 8 wells of the 96-well V bottom plate, and 10, 100, 1000, and 10,000 Jurkat cells were added into two wells each. Cells were not stained, but each well was fixed with methanol as described above. The remainder of the protocol, until the sort, proceeded as described above, and each of the 8 wells received a unique barcoded TCR reverse transcription primer. During the sort, debris and doublets were gated out, and then all cells were collected. Post-sort, after the DTT de-crosslink step, the 1.5mL tube was heated to 95°C for cell-lysis. Lysis buffer was then added to the tube and cDNA extraction and PCR proceeded as described above.

#### ELISPOT

The frequency of IFNγ secreting cells was assessed by the Johns Hopkins Immune Monitoring Core using an ELISpot assay (Mabtech, Cat. No. 3420-4HST) with responder PBMC at 2x10^5^ cells per well, KRAS peptides at 2 μg/ml in DMSO (from 4 mg/mL stock), CEF (CTL, Cat. No. PA-CEF-002) and αCD3 antibody (Mabtech, in kit) at vendor recommended concentrations. Tissue culture medium (RPMI-1640) was supplemented with 10% FBS (Hyclone, Cat. No. SH30071.03). The assays were incubated overnight (37°C, 5% CO2, >18 h) and after development were read using an iSpot spectrum reader (AID). The results underwent technical review before release to the investigator.

In Figure 2D, ELISPOT values were averaged across the four peptides for each donor and plotted against the average number of total productive KRAS-reactive sequences across the four peptides, identified by INCERTS for that donor.

#### Single-cell RNA (scRNA-seq) and TCR (scTCR-seq) sample preparation and sequencing

scRNA library preparations were performed on unstimulated PBMCs from one of the four donors, using the 10x Genomics Chromium Single Cell system and Chromium Single Cell 5′ Library & Gel Bead Kit v2 (10x Genomics, Cat. No. PN-1000263). TCRs were enriched by following manufacturer’s instructions using the TCR Amplification kit (10x Genomics, Cat. No. PN-1000252). The initial cell input was 17,000 PBMCs to recover a total of 10,000 cells. Sequencing, at a depth of 50,000 reads per cell, was performed on the NovaSeq platform (Illumina) using 10x Genomics recommended features.

### Quantification and statistical analysis

#### Software

Illumina Fastq files were demultiplexed based on sample-specific and sorted population-specific barcodes using the cutAdapt^52^ software with settings allowing for 1 mismatched nucleotide in the barcode. MiXCR^53^ version 3.0.13 was then used to identify TCR sequences from demultiplexed fastq sequencing files with the following settings: --species hs --starting-material rna --receptor-type TRB --5-end v-primers --3-end c-primers --adapters no-adapters --assemble "-OaddReadsCountOnClustering = true." All sequences with clone count ≥10 were used for further analysis. All further data processing was performed using R and Microsoft Excel. TCR sequence clustering was performed with GLIPH2^36^ using reference version 2.0, CD48, and all amino acids interchangeable for settings. The motif diagram was generated using the msa Bioconductor package in R.

#### Identifying INCERTS reactive TCR sequences

TCR sequences were considered to be reactive to a peptide if the sequence was productive, present in the population that was positive for either or both CD69 and CD137, and was not found in CD69^−^CD137^−^-population at a frequency above 1% of the total counts of the specific CDR3 amino acid clone. TCR sequences were considered to be reactive for multiple peptides if the clone count associated with that peptide was at a frequency above 1% of the total counts of the specific CDR3 amino acid clone in the corresponding activated cell population. 1% clone specific frequency cutoff was determined based on the number of Jurkat sequences that were associated with a different well-specific barcode. Although Jurkat cells received a unique TCR barcode, in the sequencing data of the activated populations, 1% of the Jurkat clone sequences were incorrectly associated with a different barcode, potentially due to chimeric amplicon formation during PCR.

#### Single-cell RNA and TCR-seq analysis

Sequences were processed using the Cellranger 5.0.1 pipeline (10x Genomics) and mapped to the human reference genome (GRCh38). The raw feature-barcode matrix was processed with Scanpy (version 1.8.1),^54^ putative cell doublets were removed using the Scrublet package,^55^ and leiden clustering was performed at a resolution of 2.0 to capture major cell types and subtypes. Differentially expressed genes across the leiden clusters were determined using the scanpy.tl.rank_genes_groups function. Clusters were manually annotated based on the RNA expression of known cell type marker genes, filtered using Besca,^56^ and confirmed using annotation pipelines including Azimuth^57^ and Celltypist.^58^ TCR repertoire analysis was performed with Scirpy (version 0.9.1)^59^ and productive TCR chain pairing status was determined with the scirpy.tl.chain_qc() function. Comparisons between INCERTS TCRβ CDR3 nucleotide sequences and single-cell TCRβ CDR3 sequences were performed using R v4.1.1. TCRβ CDR3 nucleotide sequences were considered a match to a single-cell TCRβ CDR3 nucleotide sequence if they were identical, as were J gene calls. In cases where various single-cell alpha chains were paired to the same beta chain sequence, INCERTS T cell types were considered a match if at least one of the possible alpha-beta paired phenotypes was identical.

## Data Availability

• De-identified scRNA-sequencing with matched TCR sequencing data have been deposited at dbGaP. Bulk TCR-sequencing data are available upon request. Accession numbers are listed in the key resources table. • This paper does not report original code. • Any additional information required to reanalyze the data reported in this paper is available from the lead contact upon request.

## Supplemental information

Table S1. List of productive, peptide-reactive TCRβ sequences identified using INCERTS method, related to Figure 2 Peptide, CD4 or CD8 T cell type, and cancer type corresponding to each CDR3 are listed. CDR3s reactive to more than one peptide are listed multiple times in the first part of the table, and then included again at the bottom of the table in a condensed form, where each TCRβ chain reactive to more than one peptide is listed once, with the corresponding T cell types, donor, peptide, and cancer type.

Table S2. GLIPH2 output of identified clusters of peptide-reactive, productive TCRβ CDR3 sequences identified via INCERTS, related to Figure 2C Refer to GLIPH2 publication for column meanings. Freq column values are MiXCR clone count.

Table S3. Single-cell and INCERTS sequence information for overlapping TCRβ CDR3s, related to Figure 2E Cell types and J genes match between single-cell and INCERTS. Full-length sequence information for published KRAS-reactive TCRs.

Table S4. Summary of each population; the number of sorted cells; the number of unique, productive, TCRβ CDR3 clones identified; and sequencing quality data for each population, related to Figure 2
